# The association between ADHD symptoms and different dimensions of problematic use of social media among adolescent: results from the “LifeOnSoMe”-study

**DOI:** 10.1186/s12889-026-27631-7

**Published:** 2026-05-18

**Authors:** Hilde Einarsdatter Danielsen, Turi Reiten Finserås, Tormod Bøe, Gunnhild Johnsen Hjetland, Amanda Iselin Olesen Andersen, Ian Colman, Jens Christoffer Skogen

**Affiliations:** 1 Department of Health Promotion, Norwegian Institute of Public Health, Zander Kaaes gt. 7, Bergen, 5015 Norway; 2 Department of Clinical Psychology, Faculty of Psychology, University of Bergen, Bergen, Norway; 3 Department of Psychology, Norwegian University of Science and Technology, Trondheim, NO- 7491 Norway; 4 Department of Psychosocial Science, Faculty of Psychology, University of Bergen, Bergen, Norway; 5 RKBU Vest, NORCE Norwegian Research Centre, Bergen, Norway; 6 Centre for Evaluation of Public Health Measures, Norwegian Institute of Public Health, Oslo, Norway; 7 School of Epidemiology and Public Health, University of Ottawa, Ottawa, Canada; 8 Centre for Fertility and Health, Norwegian Institute of Public Health, Oslo, Norway; 9 Center for Alcohol and Drug Research (KORFOR), Stavanger University Hospital, Stavanger, Norway

**Keywords:** Problematic social media use, ADHD symptoms, Subjective overuse, Social obligations, Source of concern, Adolescents

## Abstract

**Background:**

Over the last years, there has been a rise in ADHD symptoms among adolescents. Concerns have been raised about a potential link with problematic social media use and studies indicate a positive association between ADHD symptoms and problematic social media use. However, these studies have often assessed problematic use using crude or non-specific measures. The aim of the present study was to investigate this association using three novel dimensions of problematic social media use: subjective overuse, social obligations, and source of concern.

**Methods:**

The present study is based on a cross-sectional survey, the “LifeOnSoMe”-study (*N* = 3568), conducted in 2020 and 2021. Participants were senior high school students (mean age 17.3 years, 55.6% girls) in Bergen, Norway. Gender-specific linear regression analyses were performed with ADHD symptoms as the independent variable and three dimensions of problematic social media use (subjective overuse, social obligation and source of concern) as the dependent variable in separate analyses. The analyses were adjusted for subjective socioeconomic status and symptoms of depression.

**Results:**

ADHD symptoms were positively and significantly associated with all dimensions of problematic social media use in the fully adjusted models.

**Conclusions:**

The results from this study are in line with previous studies on the association between ADHD symptoms and problematic social media use. In addition, the conceptualization of problematic social media use applied in this study points to potential differences in strength of association between ADHD symptoms and various aspects of social media use.

## Introduction

Social media is integral in our society, being a particularly important part of the daily life among adolescents. Social media use has increased in recent years, and in 2023, there were 4.9 billion social media users globally [55]. In Norway, nine out of ten of the population use social media, and adolescents are particularly avid users [59]. At the same time as we have seen a rise in social media use, there has also been a rise in mental health problems [35], including attention-deficit/hyperactivity (ADHD) diagnoses among adolescents in Norway [10]. Some have pointed to social media as a potential source for the rise in mental health problems [35], and there are increased concerns about whether social media use has led to an increase in ADHD symptoms (e.g.; [17, 46, 68]).

ADHD is a neurodevelopmental disorder that persists throughout life or a great part of a person’s life and where the core symptoms are inattention, impulsivity, and hyperactivity [1]. Some have hypothesized that digital media use may exacerbate ADHD symptoms by allowing for passive or reactive attentional patterns in response to rapidly changing stimuli, which could hinder the development of attentional skills [32]. Longitudinal studies have found support for this hypothesis for problematic digital media use [51], time spent using social media [39], and for problematic social media use [17]. However, a systematic literature review suggests that the relationship between ADHD symptoms and digital media is likely reciprocal [62]. Social media may be a source of immediate and quick rewards [54], which adolescents with ADHD are more vulnerable to [5]. Social media may serve as an attractive external distractor to adolescents [13], especially to adolescents who exhibit attention deficits, impulsivity, and hyperactivity, and for some this could lead to an increased risk of developing problematic social media use [60].

Research has reported an association between problematic social media use and ADHD symptoms (e.g. [3, 7, 17, 22]). For instance, Arness and Ollis [7], found a significant positive association between attention dysregulation and problematic social media use, and that this association was mediated by impulsive use. They also found that attention dysregulation was negatively associated with other types of use, such as using social media for seeking information and entertainment [7]. Another study found a positive association between both hyperactivity and inattention with problematic social media use [42]. Furthermore, a study relying on both parent- and self-report found that intense social media use was moderately and positively associated with impulsivity and hyperactivity [12].

However, most of the studies on problematic social media use and ADHD symptoms have relied on addiction criteria or merely the amount of time spent on social media to define problematic use (e.g. [4, 12, 17, 65]),. While time spent on social media has often been considered a key factor (e.g., Schønning et al., [53], there is now a growing consensus that time alone is not a serviceable measure of problematic use. Also, addiction criteria have been criticized when used as part of behavioral addictions [15, 30]. Specific to problematic social media use, the criteria have faced criticism for possibly labeling normal behavior as pathological [21] and for including elements that are unrelated to psychopathological symptoms [26]. Therefore, it is necessary to approach problematic social media use with criteria moving beyond addictive measures defined for other behaviors [20], and instead consider other factors, such as motivations, engagement and attitudes concerning social media use [7]. Thus, Finserås et al., [24] used an exploratory approach to re-investigate the concept of problematic social media use among adolescents, by using a survey based on qualitative focus-group interviews with Norwegian senior high school students and their experiences using social media [31]. From the survey data, Finserås et al., [24] identified three dimensions of problematic social media use: subjective overuse, social obligations, and concern. Subjective overuse entails feelings of using social media too much and parental concern about the adolescents’ social media use [24]. Social obligations covers feelings of obligations to be present on social media and to follow social norms on social media, as well as the fear of missing out on something perceived as socially important (the concept ‘Fear of Missing Out’ (FOMO; [24, 49]). Finally, source of concern reflects subjective feelings of being overwhelmed and concerned about their own social media use [24]. All three factors have been found to be positively associated with mental health problems [24].

This study aims to examine the association between ADHD symptoms and problematic social media use among adolescents, using three different dimensions of problematic social media use. By using novel dimensions of problematic social media use based on adolescents’ own perspectives and experiences, the study will address an overlooked aspect in the research literature and expand our understanding of this relation. We hypothesised that ADHD symptoms would be positively associated with (i) subjective overuse, (ii) social obligations, and (iii) source of concern. Depression was included as a covariate as it is a common comorbid disorder for individuals with ADHD [16], and has been shown to be associated with problematic social media use [6, 33]. Previous research has also shown that socioeconomic status is associated with both social media use [56, 61] and ADHD symptoms [52], so this was also included as a control variable.

## Methods

### Data collection

The present study is based on data from the “LifeOnSoMe”-study carried out at public senior high schools in Bergen, Norway [31, 57]. Students 16 years or older were invited to participate. Information about the survey was given both by the teachers and digitally, and the survey itself was conducted digitally. One school hour was set aside for carrying out the survey. The data used in the present study was collected in September-October 2020 and in June-September 2021. All the participants who responded in the first wave were also invited for the second wave. The response rates were 53.0% in 2020 and 35.4% in 2021. The samples were combined, and for the participants responding both in 2020 and 2021, the response set from 2020 was retained. The total number of respondents was 3771. A total of 203 respondents were excluded from the dataset due to missing age (*n* = 157), missing gender (*n* = 5), and those reporting other/non-binary gender were removed from the final dataset due to the small number (*n* = 41). Mean age was 17.3 (standard deviation 1.0), and 55.6% of the participants were girls. The final sample consisted of 3568 participants.

### Study variables

#### Symptoms of attention/deficit hyperactivity disorder (ADHD)

Symptoms of Attention Deficit/Hyperactivity Disorder (ADHD) served as the predictor variable and was assessed using the screening questionnaire of the Adult ADHD-Self-Report Scale (ASRS; [34]) and was for the purpose of the present study used as a continuous measure. Both the full ASRS and the six-item version demonstrate good psychometric properties in clinical and population samples [19, 64]. The translated Norwegian version also shows excellent psychometric properties [19]. Both versions are considered reliable for ADHD assessment and align with clinical diagnostic criteria such as DSM [1, 19, 66].The response categories were “never” (scored 1), “rarely” (scored 2), “sometimes” (scored 3), “often” (scored 4), and “very often” (scored 5). A total score was created by taking the mean of the six items, with a range from 1 to 5. Higher scores indicate higher levels of ADHD symptoms. In this study sample, the McDonald’s Omega was 0.83 for the ASRS, indicating a high level of internal consistency.

#### Problematic social media use

Problematic social media use served as the dependent variables, and was assessed with three factors named subjective overuse, social obligations, and source of concern. The factors were identified in a previous study from the same dataset [24], and the original items were based on qualitative interviews with adolescents [31]. For all items, there were five response options: “not at all” (scored 1), “relatively little” (scored 2), “sometimes/in part” (scored 3), “a great deal” (scored 4), and “a lot” (scored 5). Higher scores indicate more problematic social media use related to the respective factors. See appendix A for an overview of items.

- *Subjective overuse* was assessed by five items. The total score was obtained by summing the individual score of each item and yielded a potential maximum score of 25. McDonald’s Omega coefficient was 0.83, indicating a high level of internal consistency [40].
- *Social obligations* were assessed by eight items. The total score was obtained by summing the individual score of each item and yielded a potential maximum score of 40. McDonald’s Omega coefficient was 0.85, indicating a high level of internal consistency [40].
- *Source of concern* was assessed by three items. The total score was obtained by summing the individual score of each item and yielded a potential maximum score of 15. McDonald’s Omega coefficient was 0.66, indicating minimally acceptable internal consistency [40].

#### Age and gender

Age and gender were self-reported. For gender, the participants could choose between three options: “girl”, “boy”, and “other/non-binary”. Too few participants reported “other/non-binary” (*n* = 41) and were therefore excluded from the dataset due to privacy concerns.

#### Subjective socioeconomic status

Subjective socioeconomic status was measured by single item asking the participants to estimate how economically well off they consider their family to be compared to others, ranging from “very poor” (scored 0) to “very well off” (scored 10). A similar question format has been used with adolescents in other samples to determine subjective socioeconomic status ([28]; see review in [50]). Previous studies in Norwegian samples have also found this item to align relatively well with more objective measures of social status such as household income obtained from tax-registries and parental work affiliation [14].

#### Symptoms of depression

Symptoms of depression was assessed by using the Short Mood and Feelings Questionnaire (SMFQ; [41]) self-report and was for the purpose of this study used as a continuous measure. The questionnaire consists of 13 items and has shown good psychometric properties in community-based samples of adolescents and young adults [23, 63]. The response categories were “not true” (scored 0), “sometimes true” (scored 1), and “true” (scored 2). In this study sample, the SMFQ demonstrated a McDonald’s Omega of 0.91, indicating a high level of internal consistency.

### Ethical considerations

Participants were 16 years or older and consented to participation themselves. The participants gave informed consent by agreeing and continuing to the survey online. The study was conducted in accordance with the guidelines of the Declaration of Helsinki and with the General Data Protection Regulation and approved by the Regional Ethical Committee (REK) in Norway (REK#65611). Additional information about the study is available in previous publications [31, 56].

### Statistical analyses

Preliminary analyses showed a gender interaction effect between ADHD symptoms and dimensions of problematic social media use; therefore, the following analyses were performed stratified on gender. Descriptive statistics stratified on gender were calculated for nominal and ordinal variables in terms of distribution. Correlational analyses stratified on gender for the study variables ADHD symptoms, subjective socioeconomic status, and symptoms of depression were performed (see Appendix B and C). The summed mean score for ADHD symptoms were divided into gender-specific quartiles to examine ADHD symptoms across varying symptom intensities and identifying a potential dose-response relationship. Also, the mean score was calculated for each of the problematic use factors (subjective overuse, social obligations, and social concern) and then transformed into Z-scores. The quartiles of ADHD symptoms were defined as the independent variable, and the Z-scores of the problematic use factors were defined as dependent variables. This was done to aid interpretation, as the beta then represents the change in standard deviations of the dependent variable across quartiles of ADHD symptoms. Subjective socioeconomic status and symptoms of depression were kept as unstandardized continuous measures. A series of linear regression analyses with each of the problematic use factors as dependent variables were calculated, where step 1 was a simple regression analysis with only quartiles of ADHD symptoms as the independent variable. In step 2, a multiple regression was computed which also included subjective socioeconomic status as a potential covariate, and in step 3, a multiple regression model was computed, which in addition included symptoms of depression as a potential covariate.

Missing data were handled by listwise deletion, ranging between 2.5% and 8.7%. The analyses were performed using the statistical program IBM SPSS Statistics 28.

## Results

In Table 1, results from descriptive analyses for the sample are presented.

**Table 1:** Descriptive data for boys and girls in the sample (*N* = 3568)

	Boys (*N* = 1596, 44.4%)	Girls (*N* = 1972, 55.6%)
	Mean (SD)	Mean (SD)
Age	17.28 (1.00)	17.29 (1.01)
Symptoms of ADHD	2.68 (0.77)	2.92 (0.77)
Subjective socioeconomic status	7.41 (1.82)	6.94 (1.78)
Symptoms of depression	4.99 (5.03)	8.93 (6.36)

*N* =3568

The average ADHD symptoms score was a little higher among the girls than the boys (2.92 vs. 2.68). Also, the girls in the sample reported more symptoms of depression than boys (8.93 vs. 4.99).

In Table 2, the results from the regression analyses including the factor subjective overuse as the dependent variable are presented.

**Table 2:** Multiple regression analyses predicting subjective overuse for boys and girls

		Boys									Girls								
		Model 1 (*n* = 1504)			Model 2 (*n* = 1483)			Model 3 (*n* = 1483)			Model 1 (*n* = 1920)			Model 2 (*n* = 1905)			Model 3 (*n* = 1905)		
		B*	SE	95% CI	B*	SE	95% CI	B*	SE	95% CI	B*	SE	95% CI	B*	SE	95% CI	B*	SE	95% CI
Symptoms of ADHD	Quartile 1 (ref.)	-	-	-	-	-	-	-	-	-	-	-	-	-	-	-	-	-	-
	Quartile 2	.34***	.07	.21,.48	.34***	.07	.20,.48	.31***	.07	.17,.45	.35***	.06	.24,.47	.36***	.06	.24,.47	.35***	.06	.24,.47
	Quartile 3	.58***	.07	.44,.71	.58***	.07	.44,.72	.52***	.07	.38,.66	.42***	.06	.31,.54	.45***	.06	.33,.57	.44***	.06	.32,.57
	Quartile 4	.75***	.07	.62,.89	.76***	.07	.63,.90	.63***	.08	.48,.78	.58***	.06	.48,.71	.62***	.06	.50,.74	.61***	.07	.47,.74
Subjective socioeconomic status					.01	.01	−.02,.04	.02	.01	−.01,.05				.03**	.01	.01,.06	.03**	.01	.01,.06
Symptoms of depression								.02***	.01	.01,.03							.00	.00	−.01,.01

*B* Beta *SE* Standard error, *CI* Confidence interval

*N* = 3568. *p<.05, **p<.01, ***p < .001

The full model containing all predictors (model 3 was statistically significant and explained 8.9% (R^2^=0.089, F(5,1482) = 30.013, *p*<.001) of the variance in subjective overuse for boys. The full model containing all predictors (model 3 was statistically significant and explained 5.4% (R^2^=0.054, F(5,1904) = 22.927, *p*<.001) of the variance of subjective overuse for girls

For both boys and girls, ADHD symptoms showed a statistically significant and positive association with subjective overuse across quartiles compared to quartile 1 (*p*-values < .001 across quartiles). The point estimates (beta) increased across quartiles, indicating that higher levels of ADHD symptoms were associated with higher levels of subjective overuse. In model 2, ADHD symptoms still showed a statistically significant and positive association with subjective overuse (*p*<.001) across all quartiles, meaning that adjusting for subjective socioeconomic status did not affect the estimated association. The association with subjective overuse remained significant for both boys and girls after the inclusion of symptoms of depression in the fully adjusted model 3 (*p*<.001).

In Table 3, the results for regression analyses including the factor social obligations as the dependent variable are presented.

**Table 3:** Multiple regression analyses predicting social obligations for boys and girls

		Boys									Girls								
		Model 1 (*n* = 1509)			Model 2 (*n* = 1487)			Model 3 (*n* = 1487)			Model 1 (*n* = 1922)			Model 2 (*n* = 1906)			Model 3 (*n* = 1906)		
		B*	SE	95% CI	B*	SE	95% CI	B*	SE	95% CI	B*	SE	95% CI	B*	SE	95% CI	B*	SE	95% CI
Symptoms of ADHD	Quartile 1 (ref.)	-	-	-	-	-	-	-	-	-	-	-	-	-	-	-	-	-	
	Quartile 2	.16*	.06	.03,.28	.15*	.06	.02,.27	.11	.06	−.02,.23	.17**	.06	.05,.29	.18**	.06	.07,.30	.13*	.06	.02,.25
	Quartile 3	.33***	.06	.20,.45	.33***	.06	.21,.45	.25***	.07	.12,.38	.28***	.06	.16,.40	.30***	.06	.18,.42	.21***	.06	.08,.33
	Quartile 4	.46***	.06	.34,.58	.47***	.06	.34,.59	.30***	.07	.16,.43	.42***	.06	.31,.54	.44***	.06	.32,.56	.28***	.07	.14,.41
Subjective Socioeconomic Status					.01	.01	−.01,.04	.02	.01	−.00,.05				.04**	.01	.01,.06	.05***	.01	.02,.07
Symptoms of depression								.03***	.01	.02,.04							.02***	.00	−.01,.03

*B* Beta, *SE* Standard error, *CI* Confidence interval

*N* = 3568. *p<.05, **p<.01., ***p < .001

The full model containing all predictors (model 3 was statistically significant and explained 5.7% (R^2^=0.057, F(5,1486) = 18.897, *p*<.001) of the variance of social obligations for boys. The full model containing all predictors (model 3 was statistically significant and explained 4.4% (R^2^=0.044, F(5,1905) = 18.426, *p*<.001) of the variance of social obligations for girls

For boys, ADHD symptoms showed a statistically significant and positive association with social obligations across quartiles compared to quartile 1 (*p*-values < .05 for quartile 2, < .001 for quartiles 3 and 4). The point estimates (beta) increased across quartiles. This indicates that higher levels of ADHD symptoms are associated with higher levels of social obligations. In model 2, ADHD symptoms still showed a statistically significant and positive association with social obligations across all quartiles (*p*<.05 for quartile 2, *p*<.001 for quartiles 3 and 4). Inclusion of symptoms of depression in the fully adjusted model 3, did however reduce the estimated association a little, and only the two highest quartiles of ADHD symptoms remained statistically significant and positively associated with social obligations (*p*<.001). For girls, ADHD symptoms showed a statistically significant and positive association with social obligations for all quartiles compared to quartile 1 (p-values < .01 for quartile 2, < .001 for quartiles 3 and 4). As for boys, the point estimates (beta) increased across quartiles. In model 2, ADHD symptoms still showed a statistically significant and positive association with social obligations across all quartiles (*p*<.01 for quartile 2, *p *< .001 for quartiles 3 and 4). Inclusion of symptoms of depression in the fully adjusted model 3, did however reduce the estimated association a little, but all quartiles of ADHD symptoms remained statistically significant and positively associated with social obligations (*p*<.05 for quartile 2, and *p*<.001 for quartiles 3 and 4).

In Table 4, the results from multiple regression analyses including the factor source of concern as the dependent variable are presented.

**Table 4:** Multiple regression analyses predicting source of concern for boys and girls

		Boys									Girls								
		Model 1 (*n* = 1476)			Model 2 (*n* = 1457)			Model 3 (*n* = 1457)			Model 1 (*n* = 1884)			Model 2 (*n* = 1870)			Model 3 (*n* = 1870)		
		B*	SE	95% CI	B*	SE	95% CI	B*	SE	95% CI	B*	SE	95% CI	B*	SE	95% CI	B*	SE	95% CI
Symptoms of ADHD	Quartile 1 (ref.)	-	-	-	-	-	-	-	-	-	-	-	-	-	-	-	-	-	-
	Quartile 2	.23***	.07	.10,.36	.23***	.07	.10,.36	.19**	.07	.06,.32	.26***	.06	.14,.38	.27***	.06	.15,.39	.22***	.06	.10,.34
	Quartile 3	.33***	.07	.20,.46	.33***	.07	.20,.47	.24***	.07	.11,.38	.32***	.06	.20,.44	.34***	.06	.22,.47	.24***	.07	.12,.37
	Quartile 4	.54***	.07	.41,.67	.53***	.07	.40,.66	.32***	.08	.18,.47	.45***	.06	.33, 58	.48***	.06	.36,.61	.31***	.07	.17,.45
Subjective socioeconomic status					−.01	.01	−.03,.02	.01	.01	−.02,.03				.04**	.01	.02,.07	.05***	.01	.03,.08
Symptoms of depression								.03***	.01	.02,.04							.02***	.00	.01,.03

*B* Beta, *SE* Standard error, *CI* Confidence interval

*N* = 3568. *p<.05, **p<.01, ***p < .001

The full model containing all predictors (model 3 was statistically significant and explained 6.5% (R^2^=0.065, F(5,1456) = 21.394, *p*<.001) of the variance in source of concern for boys. The full model containing all predictors (model 3 was statistically significant and explained 4.7% (R^2^=0.047, F(5,1869) = 19.365, *p*<.001) of the variance of source of concern for girls

For boys, ADHD symptoms showed a statistically significant and positive association with source of concern across quartiles compared to quartile 1 (*p*-values < .001 across quartiles). The point estimates (beta) increased across quartiles. This indicates that higher levels of ADHD symptoms are associated with higher levels of source of concern. Adjusting for subjective socioeconomic status in model 2 did not affect the estimated association, and ADHD symptoms still showed a statistically significant and positive association with source of concern across all quartiles (*p*<.001). Inclusion of symptoms of depression in the fully adjusted model 3, did however reduce the estimated association, but all quartiles of ADHD symptoms remained statistically significant and positively associated with source of concern (*p*<.01 for quartile 2, and *p*<.001 for quartile 3 and 4). For girls, ADHD symptoms showed a statistically significant positive association with source of concern across all quartiles compared to quartile 1 (*p*-values < .001 across quartiles). As for boys, the point estimates (beta) increased across quartiles. In model 2, ADHD symptoms still showed a statistically significant and positive association with source of concern (*p*<.001) across all quartiles. Inclusion of symptoms of depression in the fully adjusted model 3, did however reduce the estimated association a little, but all quartiles of ADHD symptoms remained statistically significant and positively associated with source of concern (*p*<.001).

Overall, and independent of gender and adjustment-level, the point estimates (beta) increased across ADHD-quartiles, indicating that higher levels of ADHD symptoms are associated with the three different factors related to problematic social media use.

## Discussion

Using a sample of Norwegian high school students, the aim of the current study was to investigate if there was an association between ADHD symptoms and problematic social media use assessed by the dimensions subjective overuse, social obligations, and source of concern. The results suggested that there is a positive association between ADHD symptoms and all three dimensions of problematic social media use after controlling for subjective socioeconomic status and symptoms of depression for both boys and girls. Across quartiles, the average increase was equal to a small effect size for all three outcomes. For the difference between the upper and the lower quartile, however, the effect size was equal to a medium-to-large effect size. To aid interpretation, all the beta coefficients represent Z-scores to assist interpretability across quartiles and across the variables subjective overuse, social obligations and source of concern. Nevertheless, further research is needed to confirm these findings other settings, explore temporality and potential causality as well as underlying mechanisms.

The results from the present study are in line with previous research on the association between problematic use of social media and ADHD symptoms among adolescents [3, 7, 17, 42]. While earlier studies assessed problematic social media use with measures based on addiction symptoms, the current study’s results extend the support for a positive association between ADHD symptoms and problematic social media use while moving beyond core addiction criteria. Interestingly, the present study revealed the same pattern of results despite using different items to assess problematic social media use. This may imply that the association between ADHD symptoms and problematic social media use holds across different conceptualizations of problematic social media use.

This study is cross-sectional, meaning that we are unable to infer causality on the relationship between ADHD symptoms and problematic use of social media. It is possible that problematic social media use contributes to increased ADHD symptoms [17]. For example, the feeling of being addicted to or constantly needing to be on social media, as reflected in the subjective overuse factor, could lead to attention difficulties. Additionally, the perceived social obligation to stay active on social media may cause adolescents to sacrifice sleep in order to stay connected with friends late at night [27, 36]. This disrupted sleep, in turn, could lead to deficits in attention and increased impulsivity.

While problematic social media use may contribute to increased ADHD symptoms, it is also important to consider the possibility of a reciprocal relation. A review of longitudinal studies on digital media use and ADHD symptoms suggests that these factors may influence each other over time, rather than one solely causing the other [62]. A reciprocal perspective on the association between problematic social media use and ADHD symptoms might provide an explanation of the associations between the three factors of problematic social media use studied here and ADHD symptoms.

Subjective overuse was the dimension that showed the strongest association with ADHD symptoms for both boys and girls. The items in this factor reflect feelings of using social media too much, which may be linked to self-regulation skills, such as inhibitory control. Inhibitory control is closely linked to executive functions such as self-regulation and impulsivity [25]. Social media enables quick access to rewards [54], and therefore, as adolescents with ADHD, and possibly also adolescents with ADHD symptoms, prefer quick rewards [5], they may use social media more than other adolescents. This may in turn lead to an even stronger feeling of subjective overuse. Also, the item “social media takes away focus from more important things”, may possibly be a consequence of difficulties with response inhibition and to stop the temptation and following impulse of reaching out to the smartphone and social media for pleasure or notifications. This is supported by a mixed-methods study that found that unknowingly and impulsive use of social media was a common problem among university students [7]. In addition, the link between subjective overuse of social media and ADHD symptoms may be influenced by emotional factors and social sensitivity. For instance, for girls who reported higher levels of depression, which may make them more attuned to both external evaluations (“My parents/guardians think I spend too much time on social media”), as well as more prone to self-critical interpretations about the time they spend on social media. Adolescents with more ADHD symptoms may experience more parent-child conflicts and may therefore internalize parental disapproval as part of their subjective overuse.

The dimension social obligations pertains to the perceived obligation to be present on social media and a fear of missing out (FoMO) on something important on social media. Social media is an arena where you can establish and maintain friendships. Adolescents with ADHD often have more difficulties with social interactions [41], and their difficulties in interpreting and responding to social cues may lead to repeated negative social experiences, which in turn, could make them more cautious or anxious about perceived social obligations online. This may explain the higher scores on the social obligation items, including experiences such as fearing that a lack of likes and comments means that something is wrong socially or an urge to respond to all incoming messages.

ADHD symptoms were also positively associated with the dimension source of concern, which cover concerns about the adolescent’s own social media use and feeling overwhelmed by social media and has been compared to digital stress [24]. Though it should be noted that this factor showed the poorest internal consistency (McDonalds omega 0.66), and the interpretation of this scale should be done with caution. It has been demonstrated that individuals with ADHD report being more sensitive to stress and have difficulties coping with stress [41, 58]. Thus, adolescents with ADHD symptoms may experience concerns related to social media use stronger than others. Another component of source of concern may be linked to a feeling of lack of control through the items “I wish I could learn more about how social media affects us” and “Sometimes I feel like I’m being monitored on social media (because what I do/where I am/who I’m with, is visible)”. These items reflect an unpredictability stemming from a lack of knowledge, which may heighten stress, particularly for adolescents with ADHD who often struggle with feelings of lack of control over current or future events [41] and might also apply to adolescents with ADHD symptoms.

Impulsivity, hyperactivity, and inattention may contribute to the effects of social media use in adolescents with ADHD, potentially creating a cycle that exacerbates their symptoms. Some research suggests that increased social media use is associated with heightened impulsivity, mediated by response inhibition, which may contribute to the worsening of ADHD symptoms [67]. This implies that impulsive behaviors could lead adolescents to engage more frequently in social media, by for instance acting on external stimuli such as notifications and potentially creating a feedback loop that amplifies ADHD symptoms. A greater tendency to be distracted might also be linked to attention deficits, which has also been shown to correlate with problematic social media use [17, 46], and could further aggravate ADHD symptoms. On the other side, Boer et al. (2020) [17] found that problematic social media use was associated with an increase in ADHD symptoms over time, particularly attention deficits [17]. Such a bidirectional or reinforcing relation raises the possibility that while inattention leads to more social media use, problematic use might, in turn, further impair attention (Özkan et al. [46]. Hyperactivity has been linked to problematic social media use over time [17] as well as to the related phenomenon of problematic internet use (Augner et al., 2023). A meta-analysis found notable associations between hyperactivity and problematic internet use (including social media use), indicating that hyperactive behaviors might contribute to problematic internet use, including social media [8]. 

The interplay between impulsivity, hyperactivity, and inattention might create a reinforcing cycle regarding problematic social media use and ADHD symptoms. The above-mentioned findings [8, 17, 46, 67], might be interpreted as support for an interplay between the different dimensions of ADHD symptoms in relation to problematic social media use. In addition to reduced meta-cognitive skills and self-awareness among adolescents with ADHD [46], it may become more challenging to resist paying excessive attention to social media (e.g., social obligations), which may lead to increased usage beyond what adolescents intend. This heightened use may reduce their sense of control over social media consumption (subjective overuse), potentially causing greater anxiety or feelings of being overwhelmed by social media (source of concern).

### Strengths and limitations

The present study has some important strengths. First, the items and the wording of the items making up the dimensions of problematic social media use are based on qualitative interviews with adolescents [24, 31]. This provides items that are close to adolescents’ experiences on and motivations for using social media and hence entail the social and cultural context of their daily lives. The ecological validity may therefore be considered high. Second, the items also capture individual motivations for use in a new way and is therefore an important expansion to the already existing measurements of problematic social media use, which are mostly based on addiction criteria. Third, the items for assessing problematic social media use were developed for social media use in general, as opposed to focusing on specific platforms, and therefore they have less likelihood of being affected by changing trends in social media, such as new platforms and new functions on social media and may thus be used over a longer period. Fourth, ASRS is a validated measure for ADHD symptoms [19]. Also, the sample consists of older adolescents, which is an understudied age group regarding ADHD symptoms and problematic use of social media.

The study also has some limitations that are worth mentioning. First, as the study is cross-sectional, it cannot shed light on the potential causality or the directionality of the association between ADHD symptoms and problematic social media use. As discussed, the association may be reverse from the present study, or reciprocal. Second, even though the items of problematic social media use are based on qualitative interviews and the sample size is large, it is restricted to high school students in Bergen, Norway. Thus, the results may not be generalizable to other age groups, cultures or countries. Third, for the factor source of concern, the McDonald’s Omega coefficient indicated a minimally acceptable internal consistency, suggesting the findings related to this factor needs to be interpreted with caution, as measurement variability may affect the precision of these findings. A fourth limitation is the use of self-report both for ADHD symptoms and social media use. Self-report measures can differ from the observed degree of ADHD symptoms and social media use [43], thus clinical assessment of ADHD symptoms and objective measures for social media use, could have given other results. In addition, when both the predictor (ADHD symptoms) and the outcome variables (subjective overuse, social obligation and source of concern) are measured using the same method (self-report), it can lead to heightened correlations that do not accurately reflect the true relationships between constructs, known as the common method variance (CMV) [37, 48]. Also, adolescents with ADHD report experiencing more negative self-perception than adolescents without ADHD symptoms [11, 18], a bias that potentially can lead to inflated correlations between the severity of ADHD symptoms and the related constructs (subjective overuse, social obligation and source of concern), not because ADHD symptoms and subjective overuse, social obligation and source of concern are inherently linked but because both are influenced by the same negative self-perception. Fifth, data were collected during the COVID-19 pandemic, a period when adolescents’ social media use and overall mental distress were elevated in many countries, including Norway [9, 47, 69]. Although data collection occurred during months with relatively few restrictions, we cannot fully rule out that pandemic-related effects may have influenced adolescents’ mental health, social media behaviors or the observed associations. Moreover, the items of the problematic social media use factors used in this study contain many various aspects of social media use, but they do not address all potentially relevant aspects of problematic social media use [24].

### Implications for future research and practice

For future research, establishing the direction of the relationship between ADHD symptoms and problematic social media use will be an important step, as such studies are still few in numbers [17]. Future research should also investigate if similar results are found in younger adolescents using the same measure for problematic social media use as the present study. This can give an indication if there are developmental periods that are especially vulnerable to developing problematic social media use [45]. Another step for further research may be to investigate social media use in a clinical sample of adolescents with ADHD, to see if the results are similar to those in the present study. A further step related to research on individual factors contributing to problematic social media use, is investigating if certain social media platforms or specific affordances are more strongly associated with both ADHD symptoms and problematic use of social media [44].

As a universal implication for all adolescents, a recommendation would be to promote a healthy use of social media. Educational programs about social media have been proposed to increase adolescents’ ability to reflect on and think critically about social media and social media use [2, 29, 38]. This could be done through educational programs about social media during school hours. More specifically, considering this study´s results, an educational program could be more selectively targeted to adolescents with ADHD symptoms. Such an educational program could have a basis in the items for problematic social media use, through reflections about the adolescents’ motivations for using social media, how they use social media and how others perceive their social media use (subjective overuse), and discussions about social norms on social media and how their peers’ behavior affects their own social media use (social obligations) [2]. In addition, education about what research has shown about potential effects of social media and mental health and how social media use for some adolescents might become problematic (source of concern) could also be a part of the educational program.

## Conclusions

This cross-sectional study found that ADHD symptoms are consistently positively associated with three dimensions of problematic social media use: subjective overuse, social obligations, and source of concern. The associations remained statistically significant when adjusting for subjective socioeconomic status and symptoms of depression. The results from this study provide empirical support in line with previous research on problematic social media use, even though a direct comparison of the results must be done with caution. However, as the present study applied a set of novel dimensions of problematic social media use, closely covering the adolescents’ experiences and motivations on social media, this study serves as a contribution to the growing field of research on problematic social media use among adolescents.

## Data Availability

Explicit consent from the participant is required by the Norwegian Health research legislation and the Norwegian Ethics committees in order to transfer health research data outside of Norway. Ethics approval for this was also dependent on storing the research data on secure storage facilities located at the Norwegian Institute of Public Health, which prevent the authors from providing the data as supplementary information. Request to access these datasets should be directed to [jens.christoffer.skogen@fhi.no](mailto: jens.christoffer.skogen@fhi.no).

## Appendix A

The three factors of problematic social media use:

*Subjective overuse*:

1. Social media takes away focus from more important things.
2. I am addicted to social media.
3. My parents/guardians think I spend too much time on social media.
4. I spend too much time on social media.
5. I wish to reduce amount of time I spend on social media.

*Social obligations*:

1. I fear I might miss out on something if I’m not on social media.
2. Social media gives me a sense of control or overview of what is going on.
3. I feel that I must like and/or comment on what my friends post on social media.
4. I feel that I have to respond to all messages, “streaks” and similar things I receive.
5. If I do not respond, like or comment, then it can have negative consequences.
6. If my friends don’t like or comment what I post on social media, I start thinking something is wrong.
7. If I don’t participate on social media, I’ll fall behind.
8. I pay close attention to what my friends/boyfriend/girlfriend/family does. through social media (for example stories, Snap map…).

*Source of concern*:

1. There is so much happening on social media that I often feel overwhelmed.
2. I wish we could learn more about how social media affects us.
3. Sometimes I feel like I’m being monitored on social media (because what I do/where I am/who I’m with, is visible).

## Appendix B

**Table 5:** Correlation coefficients (Pearson Correlation) between ADHD symptoms, subjective socioeconomic status, and symptoms of depression for boys.

		1	2	3
1.	Symptoms of ADHD (a)	—		
2.	Subjective socioeconomic status (b)	− 0.216***	—	
3.	Symptoms of depression (c)	0.528***	− 0.255***	—

All coefficients are significant at ****p*<.001

## Appendix C

**Table 6:** Correlation coefficients (Pearson Correlation) between ADHD symptoms, subjective socioeconomic status, and symptoms of depression for girls

		1	2	3
1.	Symptoms of ADHD (a)			
2.	Subjective socioeconomic status (b)	− 0.176***	—	
3.	Symptoms of depression (c)	0.490***	− 0.219***	—

All coefficients are significant at ****p*<.001

## Abbreviations

ADHD: Attention Deficit/Hyperactivity Disorder
ASRS: Adult ADHD–Self–Report Scale
SMFQ: Short Mood and Feelings Questionnaire
FoMO: Fear of Missing Out
